# A novel RNA-based approach to counteract EMT

**DOI:** 10.18632/oncoscience.532

**Published:** 2021-05-07

**Authors:** Sabrina Garbo, Marco Tripodi, Cecilia Battistelli

**Affiliations:** ^1^ Department of Molecular Medicine, Sapienza University of Rome, Rome 00165, Italy; ^2^ Department of Oncohematology, Bambino Gesù Children's Hospital, Rome 00165, Italy; ^3^ National Institute for Infectious Diseases "Lazzaro Spallanzani", Rome 00149, Italy

**Keywords:** lncRNA, EMT

Epithelial to mesenchymal transition (EMT) is a key signature in both physiological processes (i.e. development, regeneration, wound healing) and in tumor metastasis [1]. While the involvement of transcription factors (e.g. SNAIL, SLUG, ZEB1/2, TWIST1/2) has been extensively explored, the contribution of epigenetic mechanisms has emerged only in the last decades [2]; furthermore, interest is growing in the role of ncRNAs (e.g. miRNAs and lncRNAs) in modulating cell plasticity.

In particular, it was reported that HOTAIR, a well-established predictor of metastasis in different solid tumors, is involved in chromatin modification, acting as a scaffold for the general chromatin modifier PRC2 complex in tumorigenesis [3, 4]; however, the mechanisms conferring specificity to the PRC2 recruitment to genomic loci during EMT was not disclosed. In the last years, we focused on the role of the lncRNA HOTAIR in relation to the EMT master transcriptional factor SNAIL. We reported that SNAIL directly interacts with HOTAIR, thus conferring the site-specificity to the recruitment of PRC2 complex on promoters of epithelial genes upon EMT induction [5]. The central mechanistic role of HOTAIR is represented by its bridging activity that allows the interaction between SNAIL and the catalytic subunit of the PRC2 complex, EZH2. Functionally, HOTAIR depletion was shown to inhibit the SNAIL repressive capacity.

Building on this evidence, we designed an RNA-based dominant negative molecular approach to counteract HOTAIR function in hepatocellular carcinoma (HCC) cells.

**Figure 1:**
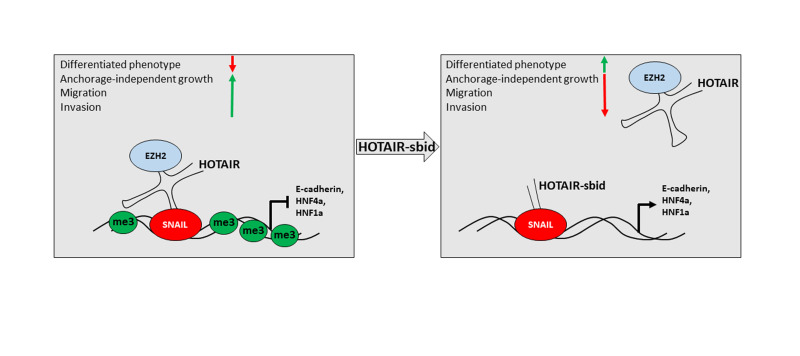
Figure 1: Schematic representation of HOTAIR-sbid activity during EMT (me3 represents H3K27me3).

RNA therapeutics represent a growing field of investigation and application. The use of RNA molecules shows several advantages since they show a very low immunogenicity, are able to penetrate the cell/nuclear membrane, and to target the desired gene even if highly expressed, they are cheap and easy to synthesize, and can be chemically modified, in order to increase their stability or to stabilize secondary structures. Moreover, concerning the delivery of these molecules, in recent years, many strategies have been developed for increasing the efficient delivery of RNA therapeutic molecules to specific target cells. Some of these strategies are represented by the use of nanoparticles, lipid nanoparticles, and extracellular vesicles, above all exosomes, properly engineered in order to increase the delivery and the on-target effects. Also other approaches have been proposed, like the conjugation of these nucleic acid molecules with sugars, lipids, peptides, nucleic acids ligands in order to interact with the cell membrane or with the surface receptors (for extensive review, see [6]). Taking advantage of a bioinformatic tool, catRAPID fragments, [7] we predicted a specific HOTAIR domain as the region with the highest affinity of interaction with SNAIL. This sequence, namely HOTAIR-sbid (for SNAIL-binding domain), devoid of the EZH2-binding capacity, was expressed in different contexts (i.e. tumor and TGFβ-induced EMT cells) to test its ability to impair endogenous HOTAIR/SNAIL pro-EMT function. Notably, HOTAIR-sbid was functionally proved to reduce cellular motility, invasiveness, anchorage-independent growth, and responsiveness to TGFβ-induced EMT. Mechanistically, while SNAIL was shown to maintain its ability to bind to its target genes, its repressive function results abolished (Figure 1). Cells not expressing HOTAIR appear, as conceivably expected, not affected by HOTAIR-sbid [8].

Overall, the described RNA-based dominant negative approach further contributes to the translational applications based on the use of RNA therapeutics. Specifically, this strategy appears conceivably devoid of off-target effects in EMT and tumorigenesis and holds promises of effectiveness when the function of lncRNAs is proved as a determinant.
